# Perioperative outcomes of robotic lobectomy for early-stage non-small cell lung cancer in elderly patients

**DOI:** 10.3389/fonc.2022.1055418

**Published:** 2022-11-28

**Authors:** Filippo Tommaso Gallina, Riccardo Tajè, Daniele Forcella, Valeria Gennari, Paolo Visca, Federico Pierconti, Cecilia Coccia, Federico Cappuzzo, Isabella Sperduti, Francesco Facciolo, Enrico Melis

**Affiliations:** ^1^ Thoracic Surgery Unit, IRCCS Regina Elena National Cancer Institute, Rome, Italy; ^2^ Department of Pathology, IRCCS Regina Elena National Cancer Institute, Rome, Italy; ^3^ Anesthesiology and Intensive Care Unit, IRCCS- Regina Elena National Cancer Institute, Rome, Italy; ^4^ Medical Oncology 2, IRCCS Regina Elena National Cancer Institute, Rome, Italy; ^5^ Biostatistics, IRCCS Regina Elena National Cancer Institute, Rome, Italy

**Keywords:** NSCLC, rats, mediastinal lymphadenectomy, elderly, postoperative complications

## Abstract

**Introduction:**

Minimally invasive surgery has become the standard for the early-stage non-small cell lung cancer (NSCLC). The appropriateness of the kind of lung resection for the elderly patients is still debated.

**Methods:**

We retrospectively reviewed patients with older than 75 years who underwent robotic lobectomy between May 2016 to June 2022. We selected 103 patients who met the inclusion criteria of the study. The preoperative cardiorespiratory functional evaluations were collected, and the risk of postoperative complications was calculated according to the Charlson Comorbidity Index, the American College of Surgery surgical risk calculator (ACS-NSQIP), EVAD score, and American Society of Anesthesiology (ASA) score. The patients were divided in two groups according to the presence of postoperative complications.

**Results:**

Forty-three patients were female, and 72.8% of the total population were former or active smokers. Thirty-five patients reported postoperative complications. The analysis of the two groups showed that the predicted postoperative forced expiratory volumes in the first second (FEV1) and forced vital capacity (FVC) were significantly lower in patients presenting postoperative complications (p=0.04). Moreover, the upstaging rate and the unexpected nodal metastases were higher in the postoperative complication groups.

**Conclusion:**

Robotic-assisted lobectomy for early-stage lung cancer is a safe and feasible approach in selected elderly patients. The factors that could predict the complication rate was the predicted postoperative FEV1 and the nodal disease.

## Introduction

Lung cancer is the leading cause of cancer-related death worldwide. The radical treatment of early-stage non-small cell lung cancer (NSCLC) is lobectomy and hilum-mediastinal lymphadenectomy (1, 2).

However, along with the improvement in life expectancy, the incidence rate of lung cancer has gradually increased among the elderly. Therefore, a growing number of patients present at diagnosis in their old age, with the pick in incidence at 75 years old (3–5). Moreover, the prevalence of frailty in NSCLC is 45% with a significant impact on survival (6).

In these patients, the new diagnosis of lung cancer is added to a significant burden of smoking-related comorbidities and chronic diseases, which impair functional reserve and may lay the ground for postoperative complications (7). To minimize the perioperative risk of complications, radical treatment is often sacrificed to reduce surgical trauma regardless of the risk of undertreatment and of a poor oncological outcome (8, 9).

In the last decades, minimally invasive surgery became the strategy of choice in the management of early-stage lung cancer, improving perioperative outcomes when compared to open surgery especially in the elderly, thus challenging the surgeon to extend radical surgery to these patients (10, 11).

In the context of minimally invasive surgery, robotic surgery is outgrowing thanks to its ability to enhance surgical maneuverability and visualization (12). Nonetheless, robotic surgery is demonstrated to improve oncological outcomes with a complication and conversion rate comparable to video-assisted surgery (13). However, the perioperative results of robotic-assisted lobectomy for early-stage lung cancer in patients older than 75 years have not been clearly assessed, and the best treatment strategy for NSCLC affecting the elderly is often omitted from guidelines and clinical trials leading to management ambiguities (9).

In order to evaluate the feasibility and the perioperative outcome of robotic lobectomy in patients with age higher than 75 years, we retrospectively analyzed our database of patients undergoing robotic pulmonary lobectomy at our institution. Clinical and pathological features affecting perioperative outcomes have been also analyzed.

## Materials and methods

In this single-center retrospective analysis, patients older than 75 years underwent robotic lobectomy for NSCLC. Data for analysis were retrieved from our lobectomy database including patients operated on from May 2016 to June 2022. Moreover, clinical charts, surgical reports, and outpatient’s clinic reports were reviewed to retrieve data about perioperative clinical and pathological characteristics and postoperative complications. General inclusion criteria for this study were patients diagnosed with NSCLC at clinical stage I and II undergoing robotic lobectomy. Patients with clinical stage III–IV were excluded; sublobar and wedge resections were also excluded.

### Preoperative assessment

In all the patients, preoperative staging was achieved through total body computed tomography (CT) and F18-fluorodeoxyglucose positron emission tomography (FDG-PET). Bone scintigraphy was performed if clinically indicated. Patients presenting with suspected hilar or mediastinal nodal metastases underwent non-invasive eco-endoscopic biopsy. To evaluate resectability and to assess preoperative cardiopulmonary function, all the patients underwent spirometry, diffusion capacity of the lung for carbon monoxide (DLCO), and arterial blood gas analysis. Postoperative predicted (ppo) forced expiratory volumes in the first second (FEV1), forced vital capacity (FVC), and DLCO values were calculated according to the anatomical techniques (14). Further or more complex functional evaluations, including 6-min walking test and cardiac stress tests, were performed in patients presenting with impaired cardiopulmonary status (14). The performance status of each patient was calculated according to the Easter Cooperative Oncology Group (ECOG) performance status scale (15). Overall risk of postoperative complication was calculated according to Charlson Comorbidity Index (16), the American College of Surgery surgical risk calculator (ACS-NSQIP), EVAD score (17), and American Society of Anesthesiology (ASA) score (18). Frailty of the enrolled patients was considered according to the modified Frailty Index (19). Before the operations, all patients had signed an informed consent to lobectomy. All patients who underwent robotic-assisted thoracoscopic surgery (RATS) operations were alerted about the possibility of conversion to thoracotomy in case of unexpected technical problems. Before the operations, all patients had a discussion in the context of a multidisciplinary meeting with the thoracic surgeon, oncologist, radiotherapist, and pneumologist.

### Surgical technique

All the patients underwent curative intended surgery by robotic-assisted lobectomy (using the Si da Vinci robot and the Xi da Vinci robot). Patients were placed in the lateral decubitus position using the single-lung ventilation with the hips fixed at the level of the table break and flexed to achieve maximum separation of the intercostal spaces. The Si da Vinci robot is positioned at the head of the patient. The Xi da Vinci robot is positioned at the back of the patient. We always proceeded performing a 3-cm utility incision at the fifth or sixth intercostal space anteriorly of the latissimus dorsi. The wound is usually protected with a soft tissue retractor. Then, we performed the other three operative ports under direct view guidance usually at the eighth or ninth intercostal space. We then started docking the robot. We always used a 30° stereoscopic robotic camera. Under direct view, the bed assistant started introducing the operative robotics arms. The lobectomy was carried out with the usual technique. The pulmonary vein, pulmonary artery, and lobar bronchus were individually isolated and divided with a vascular three-line stapler. A parenchymal stapler was also used for the division of incomplete fissures. The lobe was retrieved with an endoscopic bag. In the clinically negative-node octogenarian patients, systematic mediastinal lymphadenectomy was performed according to preoperative comorbidities, therapeutic chances, and surgical characteristics in order to avoid major complications and reduce operative time. A hilum-mediastinal lymph nodes sampling was carried out in all patients. At the end of the procedure, we usually inserted one chest tube using the camera port.

### Postoperative evaluation and follow-up

The standardize postoperative management consisted of laboratory test and chest X-ray performed at postoperative days 1 and 5 or after removing chest drain. Pleural effusion and air leakages were recorded daily and recorded in the clinical chart for each patient. Chest drainages were considered for removal when no air leakages could be detected and the pleural effusion output was <150 ml/day. Air leakages were considered prolonged when lasting more than 5 days (20). Patients presenting prolonged air leakages could be discharged with chest drainage connected to a Heimlich valve according to the patient’s preference and familiar context. Pleural effusion was considered persistent after 5 days of drainage output higher than 250 ml/day or when the drainage output was the only reason to prolong the patient in-hospital stay (21). Outpatient follow-up was performed by thoracic surgeons after 1 month from the operation. Standard follow-up consisted of chest X-ray, laboratory testing, and clinical examination. Postoperative complications were classified according to the Clavien–Dindo classification (22).

### Objectives of the study

The primary objective of the study was to report and analyze the feasibility and the complications of robotic thoracoscopic lobectomy for clinical early-stage lung cancer in a selected population of elderly patients (age ≥75 years). The secondary objective was to compare patients undergoing postoperative complications with patients presenting a regular postoperative process. The main perioperative factors that can help to predict complications in this highly selected at-high risk population have been analyzed.

### Statistical analysis

Statistical analysis was performed with SPSS 20 (IBM SPSS Statistics, IBM Corporation, Chicago, IL). Descriptive statistics was calculated and expressed as median and interquartile range (IQR).

Patients were divided in chronological order since the beginning of the thoracic robotic program in 2016 in three classes of 25 patients each and a fourth class composed of 28 patients. Postoperative complications rate has been compared among the fourth classes using χ^2^ test to determine the role of operator proficiency in the postoperative complications rate.

Intergroup analysis was performed comparing patients undergoing postoperative complications versus patients undergoing a regular clinical progress to analyses perioperative factors that may influence postoperative complications rate. The distribution of perioperative characteristics of patients in each study group was compared by using analysis of variance for continuous variables and Fisher exact test or χ^2^ test for categorical variables. Perioperative characteristics presenting statistically significant differences between the two groups (p-value < 0.05 was considered to be statistically significant) were included in the binary logistic regression model.

## Results

Out of the 954 robotic procedures performed since the beginning of the thoracic robotic program at our institution, 103 elderly patients undergoing robotic lobectomy for clinical early-stage lung cancer with an age of more than 75 years old selected. Demographic and perioperative characteristics of the enrolled population are presented in **Table 1**. A fifth of the patients were older than 80 years at surgery, and up to 70% of the patients were active or former smokers. Of the patients with previous oncological history, 32% had breast cancer, 19% had gastrointestinal cancer, and 16% had urological cancer; other previous cancers were lymphoma, laryngeal cancer, endometrial cancer, and melanoma. None of the patients had previous pulmonary resections, and one patient had thoracic radiotherapy but presented at surgery with preserved respiratory function and did not experience postoperative complications. Comprehensively, 65% of the patients enrolled presented at surgery with solitary pulmonary nodules, while the other patients had pulmonary masses larger than 30 mm. Considering pulmonary functional evaluation, spirometry and DLCO data could be retrieved for 93 patients. Ten patients had permanent tracheostomy due to previous laryngeal surgery; thus, in these patients, spirometry and DLCO were not performed. Patients presenting with ppoFEV1 or ppoDLCO below 60% were 16 and 39, respectively, and 13 patients presented with both ppoFEV1 and ppoDLCO below 60%. In these patients, 6-min walking test was performed with satisfying results. Nearly half of the patients were ASA III, and 80% of the patients presented an ACS NSQIP risk above average. The results of CCI, EVAD score, and of the other risk stratification tools are presented in **Table 2**.

**Table 1:** Demographic and perioperative characteristics of the enrolled population.

	Total (103)	Complication (35)	Regular progress (68)	p-value
**Age, median (IQR) (years)**	77 (76-79)	77 (76-79)	78 (76 - 79.3)	0.31
**Female sex, n (%)**	43 (41.7)	14 (40)	29 (42.6)	0.84
**BMI (kg/m^2^)**	26.6 (24.4 - 29)	26.6 (24.7 -28.2)	26.7 (24.3 - 29.7)	0.685
**Smoking habit, n (%)**	75 (72.8)	27 (77.1)	48 (70.6)	0.79
**Previous cancer, n (%)**	62 (60.2)	23 (65.7)	39 (57.3)	0.52
**Tumor diameter (mm)**	28 (18 - 35.3)	30 (19.5 - 37.5)	25 (16.3 - 35)	0.385
**Diameter > 30 mm, n (%)**	38 (36.9)	14 (40)	24 (35.3)	0.67
**Radiological aspect, n (%)**				0.74
** Solid**	79 (76.7)	28 (80)	51 (75)	
** Sub solid**	12 (11.7)	3 (8.6)	9 (13.3)	
** GGO**	8 (7.8)	2 (5.7)	6 (8.8)	
** Cavitary**	4 (3.8)	2 (5.7)	2 (2.9)	
**Central type**	18 (17.5)	8 (22.9)	10 (14.7)	0.41
**Clinical stage**				0.5
** IA**	39 (37.9)	14 (40)	25 (36.8)	
** IB**	37 (35.8)	11 (31.4)	26 (38.2)	
** IIA**	12 (11.7)	6 (17.2)	6 (8.8)	
** IIB**	15 (14.6)	4 (11.4)	11 (16.2)	
**FEV1 (L/min)**	2.1 (1.7 - 2.5)	2 (1.6 - 2.4)	2.2 (1.8- 2.6)	0.301
**%FEV1**	100 (86 - 121.3)	104.5 (86 - 123.8)	93 (84 - 104)	0.192
**FVC (l)**	2.8 (2.2 - 3.4)	2.5 (2.3 - 3.3)	2.8 (2.2 - 3.5)	0.566
**%FVC**	101.5 (93 - 122.5)	100 (89 - 116)	102 (93 - 124.5)	0.236
**DLCO**	15.9 (13.8 -19.4)	16.5 (13.7 - 19.7)	15.8 (13.8 - 19.1)	0.581
**%DLCO**	78 (67 - 92)	79 (66- 90)	78 (67.8 - 92)	0.936
**%ppoFEV1**	78.9 (65.5 - 94)	72.2 (64.1 - 89.2)	82.9 (67.8 - 98.1)	0.043
** %ppoFEV1 < 60%**	16 (15.5)	8 (22.9)	8 (11.8)	0.16
**%ppoFVC**	80.5 (72 - 92.6)	75.9 (66.3 - 88.4)	83.3 (74.8 - 96.5)	0.046
** %ppoFVC < 60%**	6 (5.8)	3 (8.6)	3 (4.4)	0.41
**%ppoDLCO**	63.2 (53.4 - 72)	59.7 (50.7 - 70.7)	65.2 (53.7 - 71.8)	0.449
** %ppoDLCO < 60%**	39 (37.9)	17 (48.6)	22 (32.4)	0.13
**Upper lobectomy**	53 (51.5)	19 (54.3)	34 (50)	0.84
**Operative time (min)**	229 (194 - 277.5)	240 (192 - 297.5)	194.5 (222.5 - 266.3)	0.517
**Lymphadenectomy, n (%)**				0.37
** Systematic**	32 (31.1)	14 (40)	18 (26.5)	
** Lobar specific**	36 (35)	11 (31.4)	25 (36.8)	
** None**	35 (33.9)	10 (28.6)	25 (36.8)	
**Histology, n (%)**				0.12
** Adenocarcinoma**	83 (80.6)	25 (71.4)	58 (85.3)	
**Squamous cell carcinoma**	20 (19.4)	10 (28.6)	10 (14.7)	
**pathologic stage, n (%)**				0.26
** IA**	34 (33)	10 (28.6)	24 (35.3)	
** IB**	35 (33.9)	12(34.3)	23 (33.8)	
** IIA**	11 (10.7)	3 (8.6)	8 (11.8)	
** IIB**	18 (17.5)	6 (17.1)	12 (17.6)	
** IIIA**	5 (4.9)	4 (11.4)	1 (1.5)	
**In-hospital stay (days)**	7 (5 - 8.3)	7 (5 - 8.4)	7 (5 - 8)	0.091

BMI, body mass index; DLCO, diffusion capacity of the lung for carbon monoxide; FEV1, forced expiratory volume in 1 min; FVC, forced vital capacity; ppo, predicted postoperative.

**Table 2:** Risk stratification based on multiple scores.

	Total (103)	Complication (35)	Regular progress (68)	p-value
**ASA, n (%)**				0.84
** 2**	55 (53.4)	18 (51.4)	37 (54.4)	
** 3**	48 (46.6)	17 (48.6)	31 (45.6)	
**ACS-NSQIP**				
**Any complications**	14,6 (12.1 - 19.8)	14.6 (10.5 - 19.6)	14.7 (12.5 - 19.8)	
**Any complications > 9.6%**	83 (80.6)	28 (80)	55 (80.9)	0.88
**Serious complications**	13.9 (10.4 - 18.2)	13.6 (9.4 - 17.7)	13.9 (10.6 - 18.6)	
**Serious complications > 8.3%**	87 (84.5)	29 (82.9)	58 (85.3)	0.78
**Charlson Comorbidity Index, n(%)**				0.81
** 5**	27	11	16	
** 6**	29	10	19	
** 7**	21	6	15	
** ≥8**	26	8	18	
**Modified Frailty Index, n (%)**				0.39
** 1**	27	8	19	
** 2**	31	13	18	
** 3**	19	8	11	
** ≥4**	26	6	20	
**EVAD score**				0.48
** 3-4**	33	14	19	
** 5-6**	29	9	20	
** 7-8**	19	7	12	
** ≥9**	22	5	17	
**TcRCRI**	3	1	2	1

ASA, American Society of Anesthesia; TcRCRI, Revised Cardiac Risk Index.

**Figure 1:**
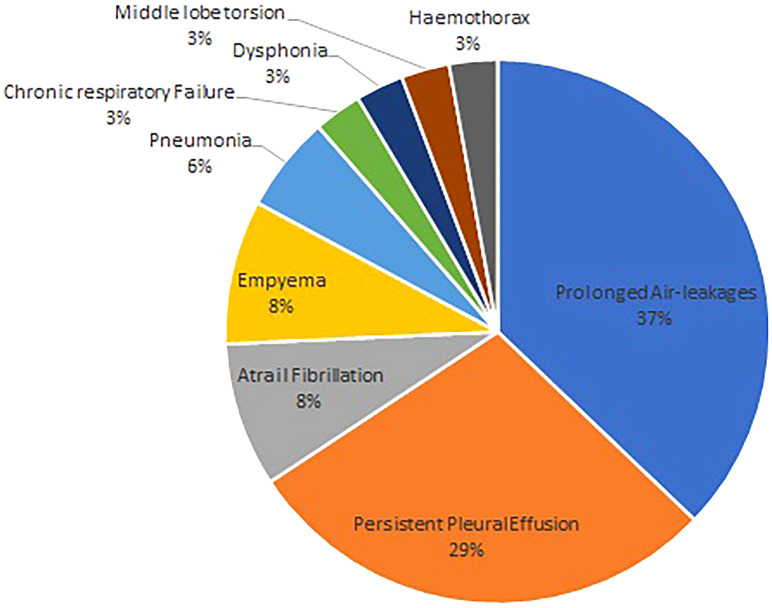
Distribution of the different postoperative complications.

The most frequent surgical procedure performed was left upper lobectomy. No intraoperative complications were recorded. At pathological examination, 80% of the patients had adenocarcinoma, and radical resection was achieved in all the enrolled patients. Nodal assessment, either through radical lymphadenectomy or lobo-specific lymphadenectomy, was performed in 52 patients. Unexpected hilar nodal metastases were found in nine patients, while two patients had metastases in both hilar and mediastinal lymph nodes. Upstaging due to postoperative minor complications were recorded in 31 patients. Major complications were recorded in four patients. Two required re-operation due to middle lobe torsion and postoperative hemothorax, respectively. One patient developed postoperative chronic respiratory failure requiring at-home oxygen administration, and one developed postoperative pleural effusion requiring thoracentesis in the outpatient clinic (**Figure 1**). No 30 days mortality was observed, and all the patients were in good clinical conditions at the 1-month reassessment.

**Figure 2:**
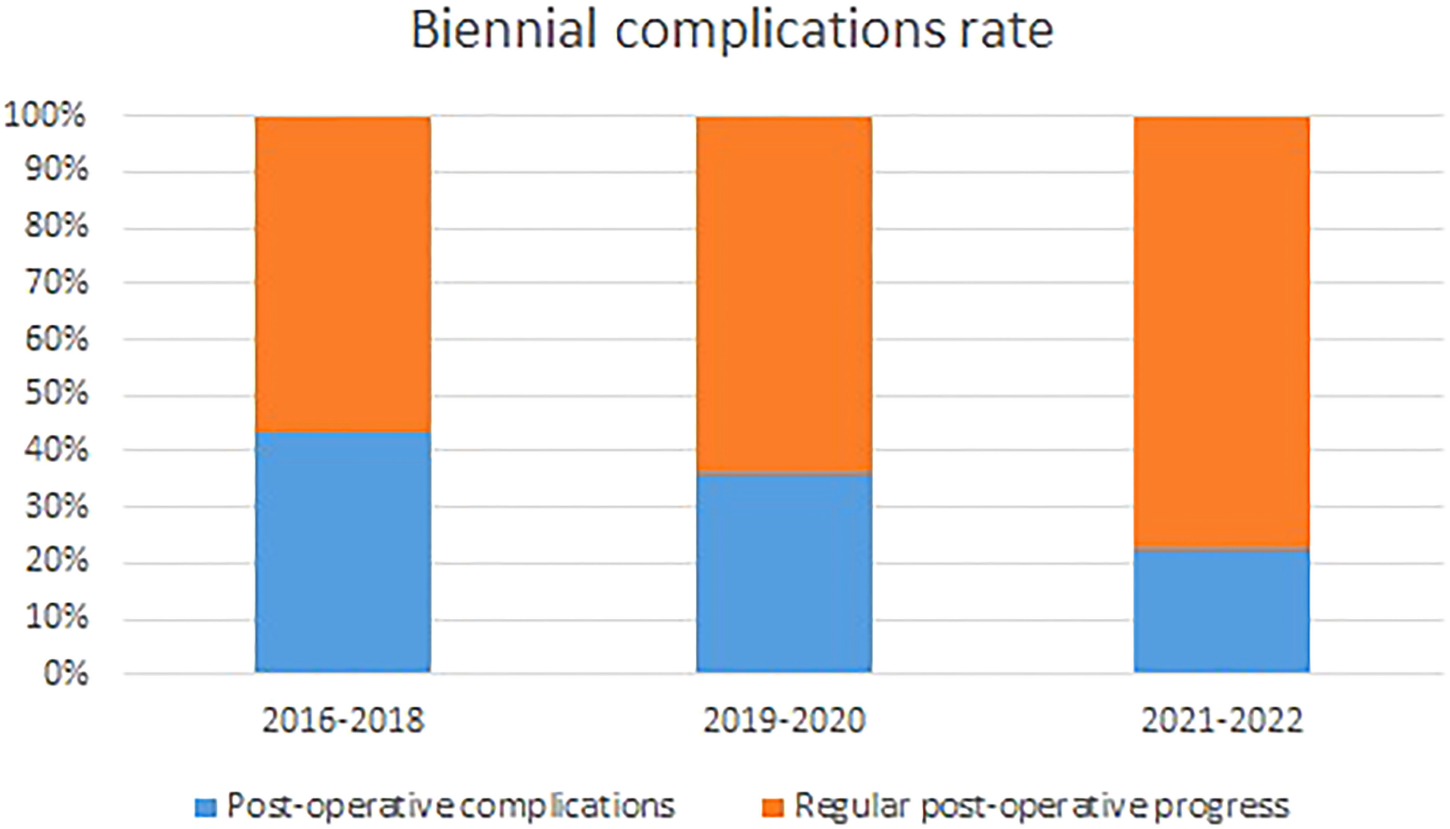
Distribution of the postoperative complications according the biennium.

As proficiency in robotic surgery has a major impact on robotic lobectomy perioperative outcome (23), patients have been stratified in chronological order since the beginning of the thoracic robotic program in three groups of 25 patients each and a fourth group of 28 patients. As shown in **Figure 2**, patients in the first two groups, operated at the beginning of the robotic thoracic program, had a 42% complications rate, while complications rate in the last two groups, including patients operated later in thoracic robotic program, dropped to 26.4%. Despite the progressive reduction in complications rate, complications distribution in the four groups failed to achieve a statistically significant difference (p=0.4).

**Figure 3:**
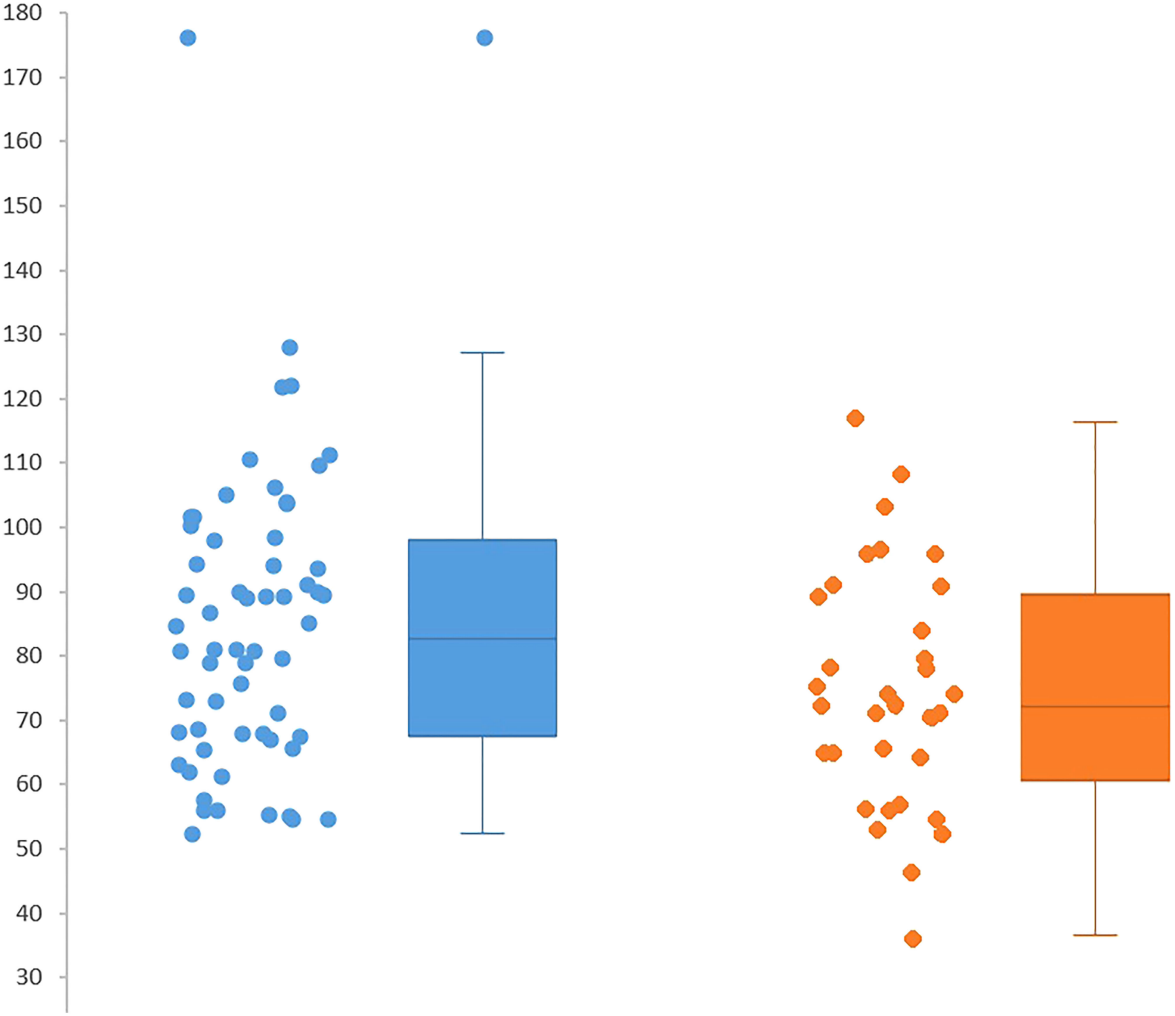
ppoFEV1 and ppoFVC evaluations in complications group vs complication group.

Perioperative characteristics of the patients undergoing postoperative complications or regular postoperative progress have been compared. As showed in **Figure 3**, at intergroup analysis, ppoFEV1 and ppoFVC demonstrated to be significantly lower in patients presenting postoperative complications (p=0.04; p=0.04). Moreover, patients in the complications group had a higher rate of pathological upstaging (p=0.006) and a higher rate of unexpected nodal metastases (p=0.04). These results remain statistically significant when only patients undergoing radical or lobar-specific lymphadenectomy have been included in the analysis. In this selected population, 10 out 25 patients undergoing postoperative complications pathological upstaging compared to 6 out 43 in the regular postoperative progress group (p=0.01) and 6 out 25 versus 2 out 43 had unexpected nodal metastases in the same groups (p=0.04). In our analysis, upstaging was mainly related to unexpected hilar or mediastinal nodal involvement determining the upstage to IIB or IIIA (locally advanced stage). In nine patients, unexpected visceral pleural invasion of peripheral nodules or radiologically undetected additional nodules in the same lobe of the primary lesion were the pathological upstaging determinant. As a consequence, the complications group had a higher rate of IIIA pathological stage when compared to the patients presenting a regular postoperative progress (p=0.04). However, no differences in the rates of systematic or lobar-specific lymphadenectomy could be found in the two groups (p=0.36). Among the analyzed perioperative characteristics, ppoFEV1, ppoFVC, pathological upstaging, and unexpected nodal metastases have been included in the binary logistic regression model (**Table 2**). Pathological upstaging was the only parameter able to predict postoperative complications (OR, 0.123; 95% CI, 0.21–0.720; p=0.02). Finally, no differences could be found in age, sex, clinical stage, tumoral diameter, NSQIP score, EVAD score, ASA score, modified Frailty Index, CCI, FEV1, FVC, DLCO, PaO2, ppoDLCO, operative time, histology, and in-hospital stay.

## Discussion

In this single-institution retrospective analysis, the perioperative outcomes of robotic pulmonary lobectomy for clinical early-stage lung cancer in patients older than 75 years were evaluated. Robotic resection could be accomplished in all the patients. Of the 103 patients, 35 patients developed postoperative complications. Minor complications were observed in 31 patients, requiring in-hospital prolongation or adjunctive pharmacological treatment, while four patients had major complications including two patients that underwent reoperation due to postoperative hemothorax and middle lobe torsion, respectively, one patient that required postoperative thoracentesis, and one patient that required at-home oxygen administration. In 65.7% of the instances, postoperative complications were prolonged air leakages and persistent pleural effusions. Both prolonged air leakage and persistent pleural effusion were treated conservatively. Pleuropulmonary infectious complications were followed with 17.1% of the complications including three patients presenting with postoperative pneumonia and three patients presenting with postoperative empyema. These patients were discharged with clinical and radiological resolution of the infection after antibiotic treatment. In patients presenting complications requiring surgical re-intervention or thoracentesis, the postoperative progress after the second surgical procedure was uneventful.

Surgical management of the elderly presenting with early-stage NSCLC has been questioned, especially as both age at diagnosis and treatment choices have radically increased through the last decades (8). The absence of univocal guidance in older patients lead to a 10% rate of undertreated elderly patients that would have benefited from an active treatment (9). In early-stage lung cancer, curative intended surgery was limited to nearly half of the patients aged 65–75 years, and the percentage further decreased in older patients (24).

Minimally invasive thoracic surgery, particularly robotic surgery, is thought to reduce surgical stress and postoperative complications still pursuing a radical intended pulmonary resection (12, 25). In the elderly, robotic pulmonary lobectomy complications rate was demonstrated to be lower than that in open surgery but similar to that in video-assisted thoracic surgery with an overall incidence approximately 30% (10, 11). These results are consistent with our findings demonstrating an overall complications rate of 33.9%. Moreover, as proficiency in robotic surgery is associated with a 15% relative reduction in 30-day overall postoperative complications (23), we evaluated the complications rate of the patients operated on in the last period of our robotic thoracic program, achieving a 25% complications rate.

Regardless of the surgeons’ proficiency, further stratification tolls may help to further reduce perioperative risk in these patients. For this reason, preoperative scores or functional test able to predict complications has been an area of interest in order to reduce surgical-related risk in patients undergoing major pulmonary resections with pre-existing impaired cardiopulmonary function or borderline age (14). Since 1973, FEV1 was identified to be a predictor of pulmonary resection tolerability, and spirometry became the standard in the evaluation of candidates for lung resection (26). Subsequently, it became evident that postoperative pulmonary function may be affected by the extension of surgical resection, and absolute preoperative FEV1 rapidly gave way to ppoFEV1 as a predictor of postoperative pulmonary complications. In this report, ppoFEV1 and ppoFVC distributions were demonstrated to be significantly lower in the postoperative complications group when compared to the regular postoperative progress group. Conversely, no differences could be found in the distributions of preoperative FEV1, FVC, or DLCO. The reduced reliability of preoperative spirometric values in minimally invasive approaches had been previously demonstrated in a retrospective analysis comparing video-assisted thoracic surgery and open thoracotomy (27). In contrast, ppoFEV1 correctly predicted postoperative complication after major pulmonary resection regardless of the surgical approach (28). According to the American College of Chest Physician guidelines, patients with ppoFEV1<60% should be referred for further cardiopulmonary function test (14). However, in our analysis, there were no differences between the two groups in the rate of patients under the ppoFEV1 <60% cutoff. As shown in **Figure 3**, according to the distributions of ppoFEV percentage values in the two groups, in this high-risk population, a more conservative cutoff at ppoFEV1 <80% may help to direct patients older than 75 years for further examinations such as cardiopulmonary exercise test or low-tech exercise test.

In the current study, both upstaging and unexpected hilar and mediastinal nodal metastases rates were higher in the postoperative complications group. Moreover, at binary logistic regression, upstaging was the only predictor of postoperative complications. Reasons underneath these results are debatable. The higher overall and nodal upstaging rates may be the results of a higher rate of lymphadenectomy that would subsequently explain the association with postoperative complications. However, no differences could be retrieved between the two groups in systematic, lobar-specific lymphadenectomy or sampling. As a reasonable explanation, pathological diagnosis of previously undetectable additional nodules, pleural infiltration, or nodal metastases may denote a more aggressive biological behavior of the disease. Therefore, the higher tumoral burden and the neoplastic lymphatic infiltration may impair pulmonary healing processes. Tumoral lymphatic invasion enhances inflammatory response, increasing microvascular permeability and eliciting pleural effusion (29). Nonetheless, malignant invasion of the thoracic lymphatic chain has been associated with the presence of substantial pleural effusion (30). Moreover, tumoral metastatic pathways have been associated with collagen deposition and pulmonary interstitial stiffness that may entail pulmonary re-expansion and enhance prolonged air leakages (31). Even if the reasons underlying the ability of upstaging and nodal upstaging in postoperative prediction may be unknown, the elderly may benefit from a more accurate hilar and mediastinal preoperative staging. On the basis of the increased complications risk, preoperative finding of pathological nodal metastases may help tailoring the best treatment strategy in this high-risk population. Nonetheless, preoperative molecular characterization may help to identify biological aggressive diseases that may benefit from a multimodal therapy.

This study has some limitations: the number of enrolled patients is limited due to the highly selective inclusion criteria; however, the application of non-parametric test in the statistical analysis may overcome this limit. Moreover, our findings demonstrated to be in trend with previous analyses presenting a similar study design in video-assisted thoracic surgery. Second, in the text, a progressive reduction in postoperative complications over time is shown. This can mostly result from an improvement in the robotic technique operators’ proficiency. This progressive improvement in patients’ perioperative outcome may have mitigated the effects of the parameters analyzed in the paper. However, when the comparison of complications rate in the stratified groups has been performed, no significant differences were retrieved, and since the beginning of our thoracic robotic program in 2016, the inclusion criteria for robotic lobectomy were consistent with no significant variations neither in the oncological nor in the functional assessment. Finally, due to the retrospective nature of the report, specific frailty evaluations or geriatric tools, other than the modified Frailty Index, to stratify high-risk patients could not be included in the analysis. Further studies are necessary to evaluate the benefits of specific frailty evaluations or geriatric tools as complementary exams to a strict pulmonary function evaluation, including predicted postoperative values and a more accurate preoperative staging.

## Conclusion

Robotic-assisted lobectomy for early-stage lung cancer was demonstrated to be a safe and feasible treatment strategy in elderly patients. The analysis of the factors that can predict the complication rate in this specific surgical populations showed that the predicted postoperative FEV1 and the preoperative staging have to be carefully evaluated to help reduce postoperative complications. In the literature, there are no specific guidelines for the preoperative staging in the elderly population. Our results showed that the nodal disease could have an impact on the postoperative complications regardless of the kind of lymphadenectomy performed. Further studies should be done to understand how the elderly patients must be stratified preoperatively, but we believe that the risk of nodal metastasis could be considered equally to the comorbidities.

## Data availability statement

The raw data supporting the conclusions of this article will be made available by the authors, without undue reservation.

## Ethics statement

The studies involving human participants were reviewed and approved by Ethics committee of the IRCCS Regina Elena National Cancer Institute; Approval Code: 1465/21 Approval Date: 23 February 2021. The patients/participants provided their written informed consent to participate in this study.

## Author contributions

Conceptualization, FG, FF. Methodology, FG, EM, RT. Software, DF, RT. Validation, FF, FC, FP. Formal analysis, FG, RT, FC. Investigation, FC, PV. Resources, FF, EM, FC. Data curation, FG, RT. Writing—original draft preparation, FG, RT, EM, FF. Writing—review and editing, EM, FF, FC, DF. Visualization, CC, FP. Supervision, FF, EM. Project administration, CC, FF. Funding acquisition, FF. All authors contributed to the article and approved the submitted version.

## Acknowledgments

We would like to extend our deepest gratitude to the dedicated contributors over the years to our project. The authors want to thank the Scientific Direction of the IRCCS “Regina Elena” National Cancer Institute for the support to our work.

## Conflict of interest

The authors declare that the research was conducted in the absence of any commercial or financial relationships that could be construed as a potential conflict of interest.

## Publisher’s note

All claims expressed in this article are solely those of the authors and do not necessarily represent those of their affiliated organizations, or those of the publisher, the editors and the reviewers. Any product that may be evaluated in this article, or claim that may be made by its manufacturer, is not guaranteed or endorsed by the publisher.

## References

[1] Siegel RL Miller KD Jemal A . Cancer statistics, 2015. CA Cancer J Clin (2015) 65:5–29. doi: 10.3322/caac.21254 25559415

[2] Silvestri GA Gonzalez AV Jantz MA Margolis ML Gould MK Tanoue LT . Methods for staging non-small cell lung cancer, 3rd ed: American college of chest physicians evidence-based clinical practice guidelines. Chest (2013) 143(Suppl. S5):e211S–50S. doi: 10.1378/chest.12-2355 23649440

[3] Sung H Ferlay J Siegel RL Laversanne M Soerjomataram I Jemal A A . Global cancer statistics 2020: GLOBOCAN estimates of incidence and mortality worldwide for 36 cancers in 185 countries. CA Cancer J Clin (2021) 71:209–49. doi: 10.3322/caac.21660 33538338

[4] Venuta F Diso D Onorati I Anile M Mantovani S Rendina EA . Lung cancer in elderly patients. J Thorac Dis (2016) 8(Suppl 11):S908–14. doi: 10.21037/jtd.2016.05.20 PMC5124601 27942414

[5] de Groot PM Wu CC Carter BW Munden RF . The epidemiology of lung cancer. Transl Lung Cancer Res (2018) 7(3):220–33. doi: 10.21037/tlcr.2018.05.06 PMC6037963 30050761

[6] Komici K Bencivenga L Navani N D'Agnano V Guerra G Bianco A . Frailty in patients with lung cancer: A systematic review and meta-analysis. Chest (2022) 162(2):485–97. doi: 10.1016/j.chest.2022.02.027 35217002

[7] Whitson BA Groth SS Duval SJ Swanson SJ Maddaus MA . Surgery for early-stage non-small cell lung cancer: A systematic review of the video-assisted thoracoscopic surgery versus thoracotomy approaches to lobectomy. Ann Thorac Surg (2008) 86(6):2008–16. doi: 10.1016/j.athoracsur.2008.07.009 19022040

[8] Bertolaccini L Viti A Terzi A . "Old people suffer the ravages of the years": Changes of treatments in elderly patients with early stage non-small cell lung cancer. Ann Transl Med (2015) 3(9):114. doi: 10.3978/j.issn.2305-5839.2015.06.02 26207242 PMC4481360

[9] Lindqvist J Jekunen A Sihvo E Johansson M Andersén H . Effect of adherence to treatment guidelines on overall survival in elderly non-small-cell lung cancer patients. Lung Cancer (2022) 171:9–17. doi: 10.1016/j.lungcan.2022.07.006 35863255

[10] Chen DL Kang PM Tao SL Wu LC Li QY Tan Q . Comparative short-term outcomes of robotic-assisted surgery for older patients with non-small cell lung cancer: A propensity matched study. Updates Surg (2022) 74(3):1087–96. doi: 10.1007/s13304-021-00992-x 33538992

[11] Veluswamy RR Whittaker Brown SA Mhango G Sigel K Nicastri DG Smith CB . Comparative effectiveness of robotic-assisted surgery for resectable lung cancer in older patients. Chest (2020) 157(5):1313–21. doi: 10.1016/j.chest.2019.09.017 PMC8500998 31589843

[12] Gallina FT Tajè R Forcella D Corzani F Cerasoli V Visca P . Oncological outcomes of robotic lobectomy and radical lymphadenectomy for early-stage non-small cell lung cancer. J Clin Med (2022) 11(8):2173. doi: 10.3390/jcm11082173 35456265 PMC9025272

[13] Gallina FT Melis E Forcella D Mercadante E Marinelli D Ceddia S . Nodal upstaging evaluation after robotic-assisted lobectomy for early-stage non-small cell lung cancer compared to video-assisted thoracic surgery and thoracotomy: A retrospective single center analysis. Front Surg (2021) 8:666158. doi: 10.3389/fsurg.2021.666158 34277693 PMC8280310

[14] Brunelli A Kim AW Berger KI Addrizzo-Harris DJ . Physiologic evaluation of the patient with lung cancer being considered for resectional surgery: Diagnosis and management of lung cancer, 3rd ed: American college of chest physicians evidence-based clinical practice guidelines. Chest (2013) 143(5 Suppl):e166S–90S. doi: 10.1378/chest.12-2395 23649437

[15] Oken MM Creech RH Tormey DC Horton J Davis TE McFadden ET . Toxicity and response criteria of the Eastern cooperative oncology group. Am J Clin Oncol (1982) 5(6):649–55. doi: 10.1097/00000421-198212000-00014 7165009

[16] Charlson ME Pompei P Ales KL MacKenzie CR . A new method of classifying prognostic comorbidity in longitudinal studies: Development and validation. J Chronic Dis (1987) 40(5):373–83. doi: 10.1016/0021-9681(87)90171-8 3558716

[17] Ferguson MK Durkin AE . A comparison of three scoring systems for predicting complications after major lung resection. Eur J Cardiothorac Surg (2003) 23(1):35–42. doi: 10.1016/S1010-7940(02)00675-9 12493501

[18] Saklad M . Grading of patients for surgical procedures. Anesthesiology (1941) 2:281–4. doi: 10.1097/00000542-194105000-00004

[19] Subramaniam S Aalberg JJ Soriano RP Divino CM . New 5-factor modified frailty index using American college of surgeons NSQIP data. J Am Coll Surg (2018) 226(2):173–81. doi: 10.1016/j.jamcollsurg.2017.11.005 29155268

[20] Brunelli A Cassivi SD Halgren L . Risk factors for prolonged air leak after pulmonary resection. Thorac Surg Clin (2010) 20(3):359–64. doi: 10.1016/j.thorsurg.2010.03.002 20619226

[21] Cerfolio RJ Bryant AS . Results of a prospective algorithm to remove chest tubes after pulmonary resection with high output. J Thorac Cardiovasc Surg (2008) 135(2):269–73. doi: 10.1016/j.jtcvs.2007.08.066 18242249

[22] Dindo D Demartines N Clavien PA . Classification of surgical complications: A new proposal with evaluation in a cohort of 6336 patients and results of a survey. Ann Surg (2004) 240(2):205–13. doi: 10.1097/01.sla.0000133083.54934.ae PMC1360123 15273542

[23] Reddy RM Gorrepati ML Oh DS Mehendale S Reed MF . Robotic-assisted versus thoracoscopic lobectomy outcomes from high-volume thoracic surgeons. Ann Thorac Surg (2018) 106(3):902–8. doi: 10.1016/j.athoracsur.2018.03.048 29704479

[24] Wang S Wong ML Hamilton N Davoren JB Jahan TM Walter LC . Impact of age and comorbidity on non-small-cell lung cancer treatment in older veterans. J Clin Oncol (2012) 30(13):1447–55. doi: 10.1200/JCO.2011.39.5269 PMC3383118 22454424

[25] Nelson DB Mehran RJ Mitchell KG Rajaram R Correa AM Bassett RL Jr . Robotic-assisted lobectomy for non-small cell lung cancer: A comprehensive institutional experience. Ann Thorac Surg (2019) 108(2):370–6. doi: 10.1016/j.athoracsur.2019.03.051 31004583

[26] Lockwood P . Lung function test results and the risk of post-thoracotomy complications. Respiration (1973) 30(6):529–42. doi: 10.1159/000193062 4762247

[27] Brunelli A Monteverde M Salati M Borri A Al Refai M Fianchini A . Stair-climbing test to evaluate maximum aerobic capacity early after lung resection. Ann Thorac Surg (2001) 72(5):1705–10. doi: 10.1016/S0003-4975(01)03100-9 11722068

[28] Epstein SK Faling LJ Daly BD Celli BR . Predicting complications after pulmonary resection. preoperative exercise testing vs a multifactorial cardiopulmonary risk index. Chest (1993) 104(3):694–700. doi: 10.1378/chest.104.3.694 8365278

[29] Ferrara N . Molecular and biological properties of vascular endothelial growth factor. J Mol Med (Berl) (1999) 77(7):527–43. doi: 10.1007/s001099900019 10494799

[30] Chernow B Sahn SA . Carcinomatous involvement of the pleura: an analysis of 96 patients. Am J Med (1977) 63(5):695–702. doi: 10.1016/0002-9343(77)90154-1 930945

[31] Navab R Strumpf D To C Pasko E Kim KS Park CJ . Integrin α11β1 regulates cancer stromal stiffness and promotes tumorigenicity and metastasis in non-small cell lung cancer. Oncogene (2016) 35(15):1899–908. doi: 10.1038/onc.2015.254 PMC4833874 26148229

